# Supplementary material for: A conserved dimer interface connects ERH and YTH family proteins to promote gene silencing
Source: Nat Commun. 2019 Jan 16;10:251. doi: 10.1038/s41467-018-08273-9 (PMC6335422; doi:10.1038/s41467-018-08273-9)
Supplement: Supplementary file 1 — Supplementary Information [file 41467_2018_8273_MOESM1_ESM.pdf]

Supplementary Information

A conserved dimer interface connects ERH and YTH family proteins to promote gene silencing

Xie et al.

Supplementary Figures: 1 – 8

Supplementary Tables: 1 – 4

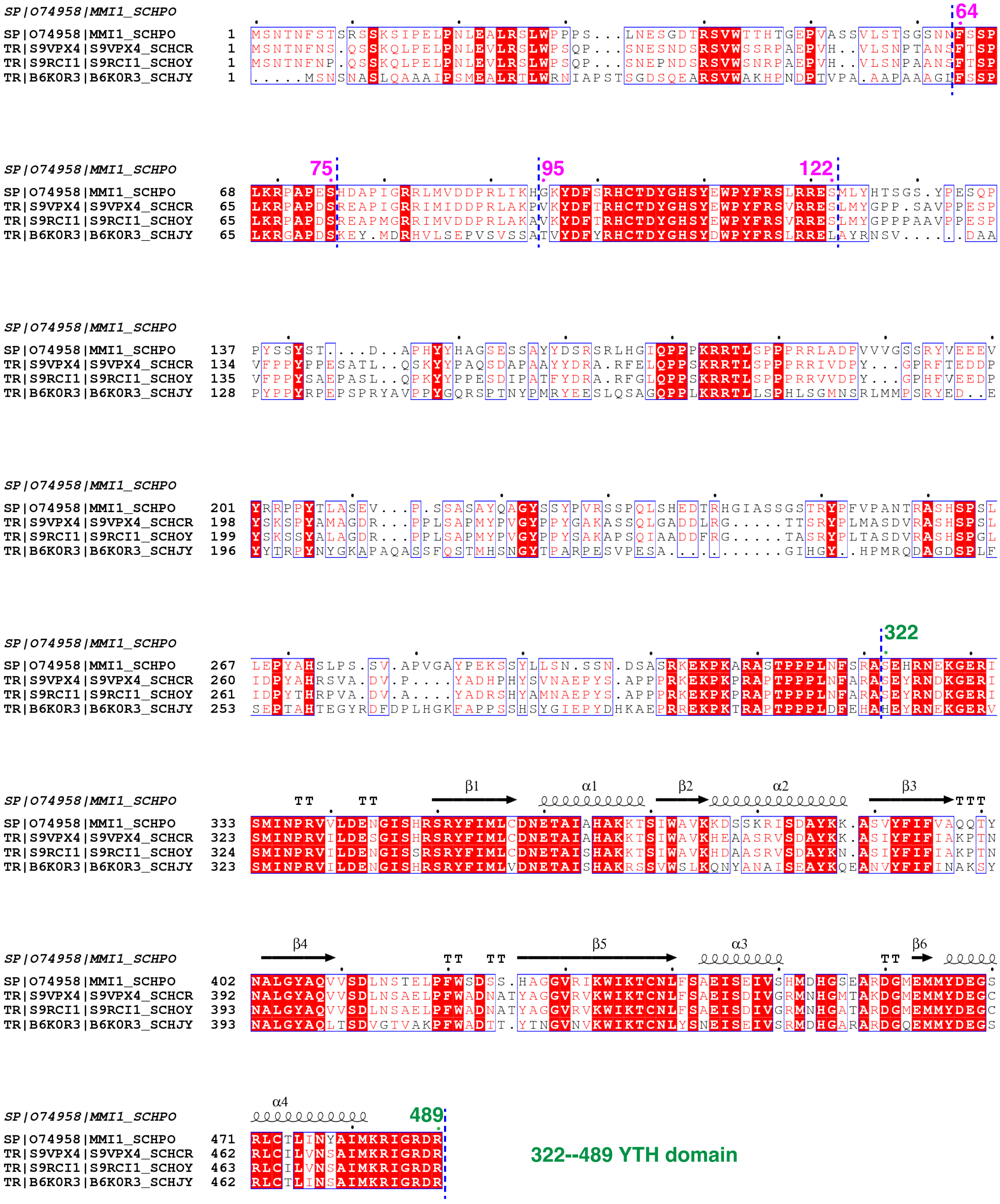

Supplementary Figure 1. Sequence alignment of Mmi1 from *S. pombe*, *S. cryophilus*, *S. octosporus* and *S. japonicus*. The alignment was generated by ESPript 3 with CLUSTALW. The secondary structure of Mmi1 YTH domain, as determined by DSSP, is shown above the sequences. The two conserved regions (residues 64-75 and 95-122) are marked by dashed lines. Aligned amino acids in red share similar biophysical properties, while those in white with red highlight are perfectly conserved.

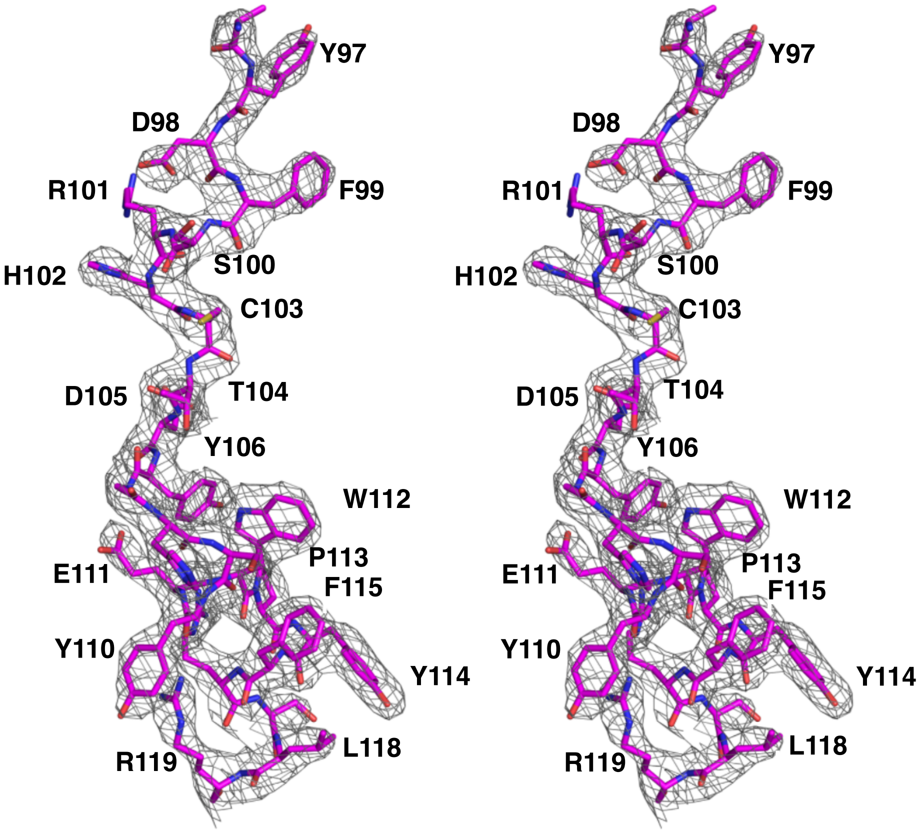

Supplementary Figure 2. Stereo view of the electron density map for Mmi1$^{96-119}$. The 2Fo-Fc electron density (gray mesh) for Mmi1$^{96-119}$ contoured at 1.2 σ is shown. Mmi1$^{96-119}$ (magenta) is shown as sticks.

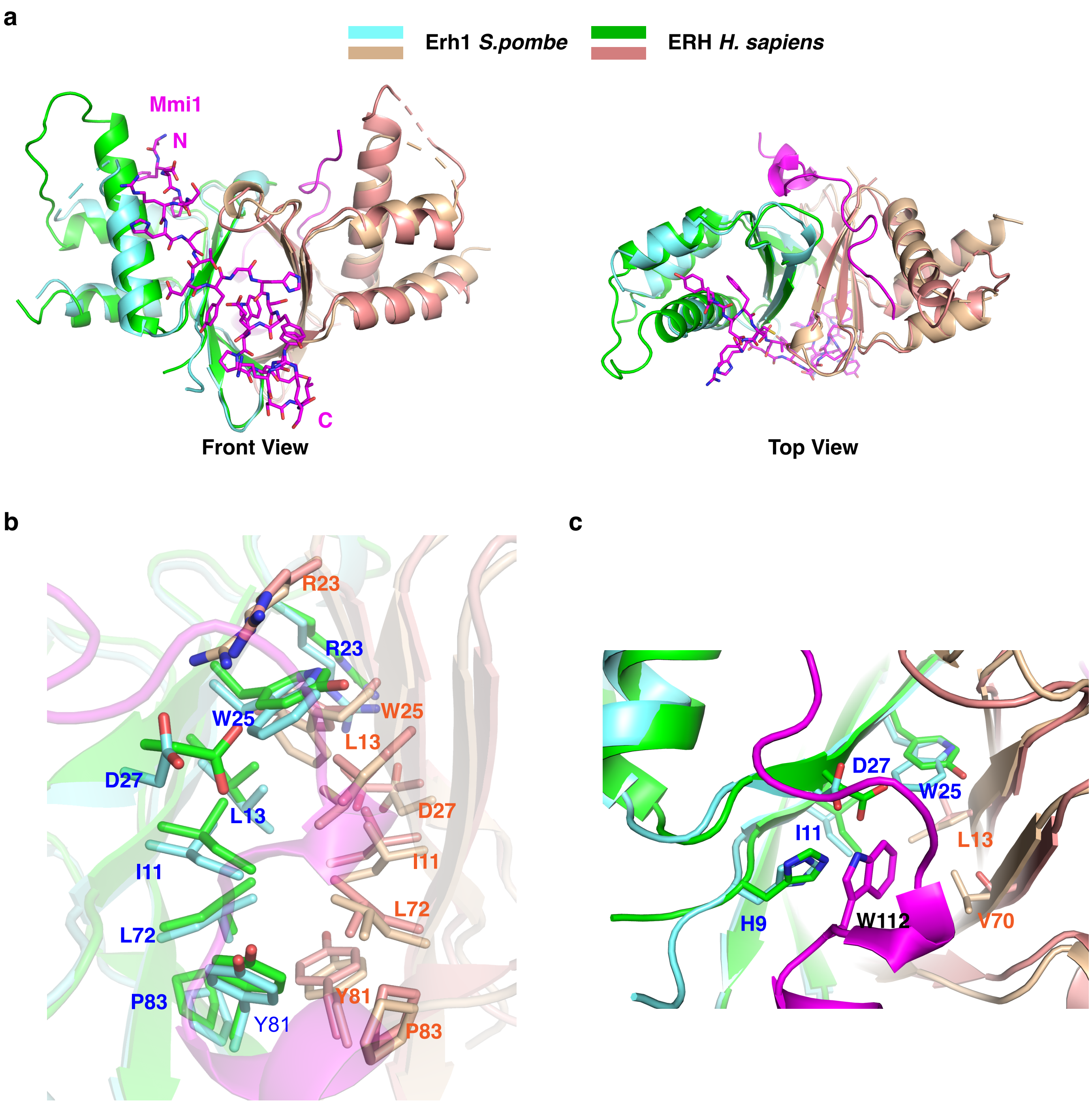

Supplementary Figure 3. Conservation of *S. pombe* Erh1 and human ERH structures. (a) Superimposition of the structures of Mmi1$^{95\text{-}122}$ bound to *S. pombe* Erh1 (shown in aquamarine and wheat colors) and human ERH (shown in green and pink colors). Front and top views are shown. **(b)** Erh1 dimer interface that interacts with Mmi1 is highly conserved in human ERH. The nearly identical conformation of amino acids in the *S. pombe* and human structures suggest that the dimer interface might be a conserved protein-protein interaction platform. **(c)** Erh1 binding pocket critical for interaction with Mmi1$^{\text{Trp112}}$ is conserved in human ERH.

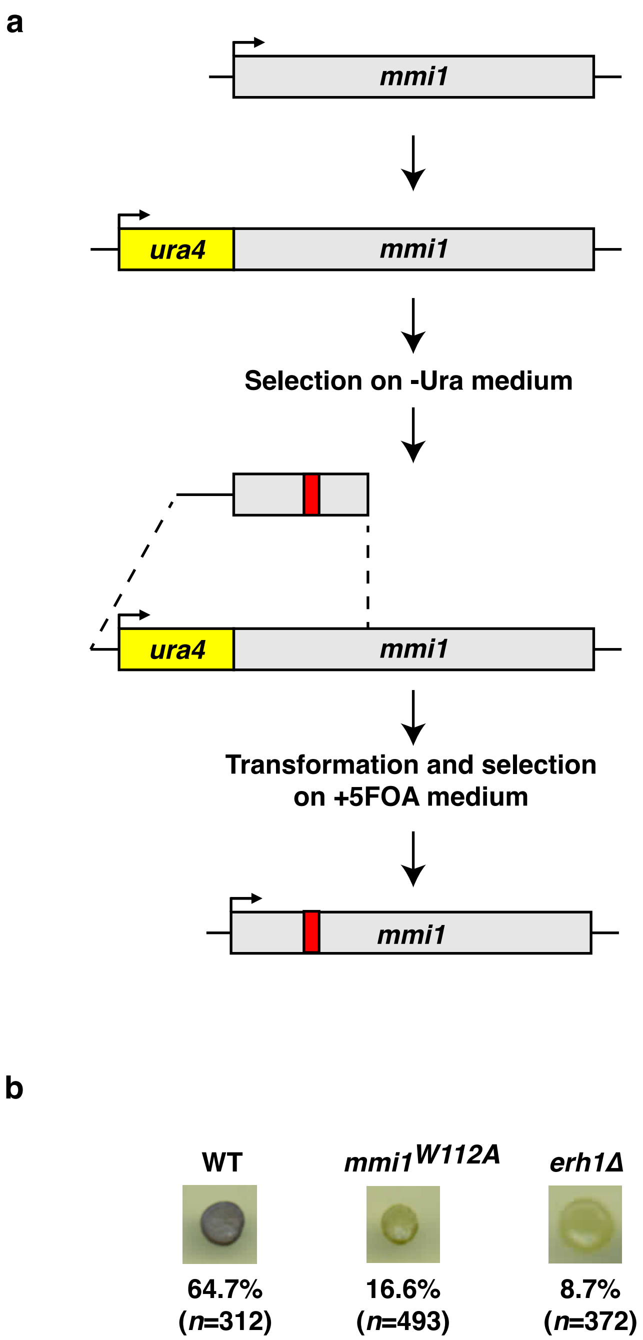

Supplementary Figure 4. Construction of the *mmi1*^{W112A} mutant allele and its impact on mating efficiency. **(a)** Construction method used to generate *mmi1*^{W112A} mutant strain. The mutation is indicated by the red rectangle. **(b)** *mmi1*^{W112A} causes mating deficiency. Cells from the indicated homothallic culture were spotted onto minimal (EMM) plates and incubated at 30°C for 3 days. The presence or absence of asci was determined by iodine staining and by microscopy. The mating efficiencies are noted (*n*, the number of cells counted in each strain).

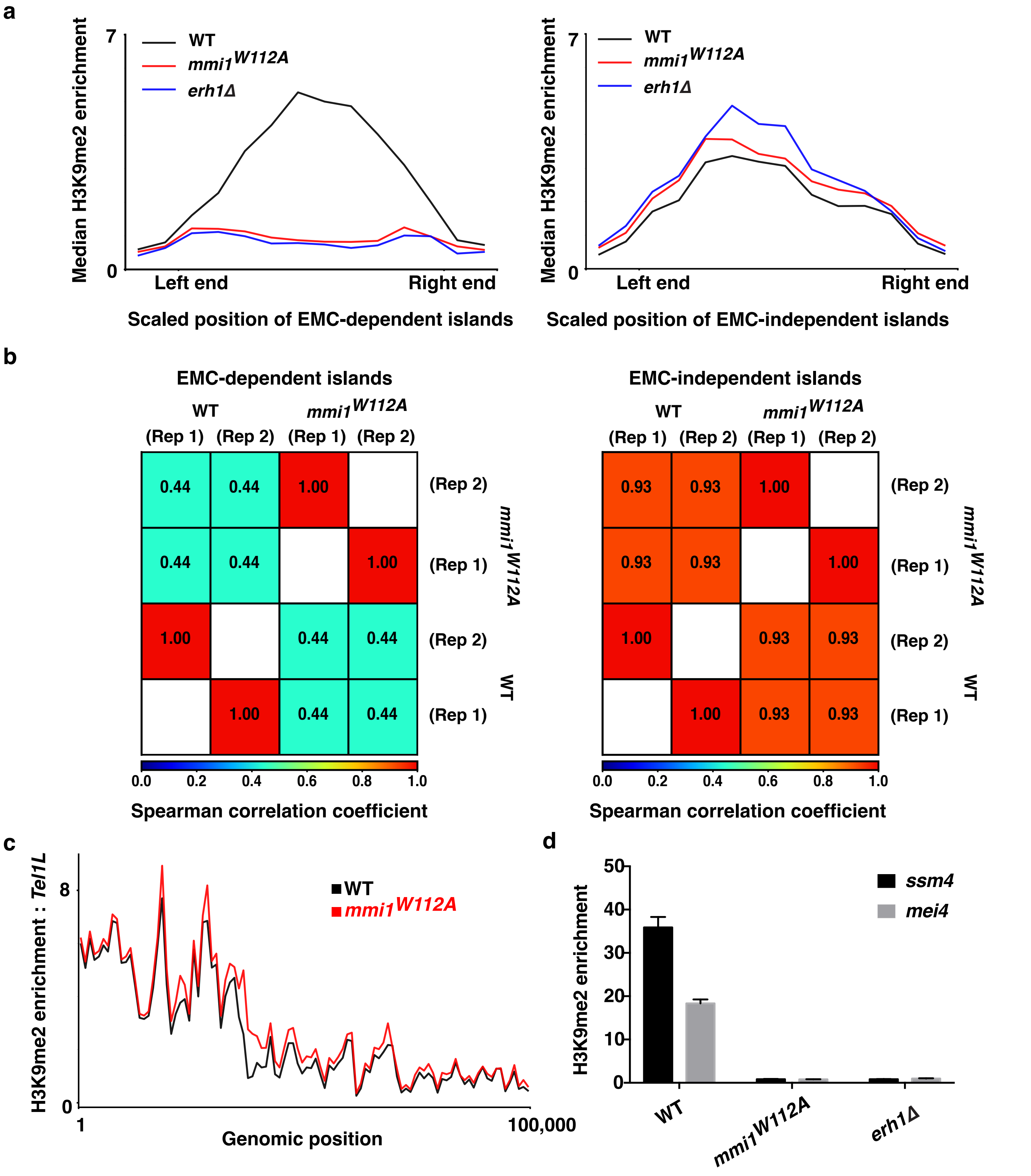

Supplementary Figure 5. Differential effects of *mmi1*^{W112A} on the assembly of EMC-dependent and -independent heterochromatin islands. **(a)** Aggregate H3K9me2 enrichment at EMC-dependent and -independent heterochromatin islands as calculated using ChIP-seq data. Shown are median H3K9me2 enrichments. The color scheme used is denoted in the upper-left corner of each plot. **(b)** Pairwise correlation heatmaps of two biological replicates of H3K9me2 ChIP-seq at EMC-dependent or -independent islands. Correlations were computed based on average H3K9me2 signals and are shown above and below the white diagonal. **(c)** H3K9me2 ChIP-seq enrichment profile at telomere 1 (left), from chromosomal positions 1 – 100,000 in WT (black) and *mmi1*^{W112A} (red) cells. **(d)** ChIP-qPCR of H3K9me2 fold enrichment at *mei4* and *ssm4* loci relative to the control *leu1* gene. Shown are mean ± SD for two independent experiments.

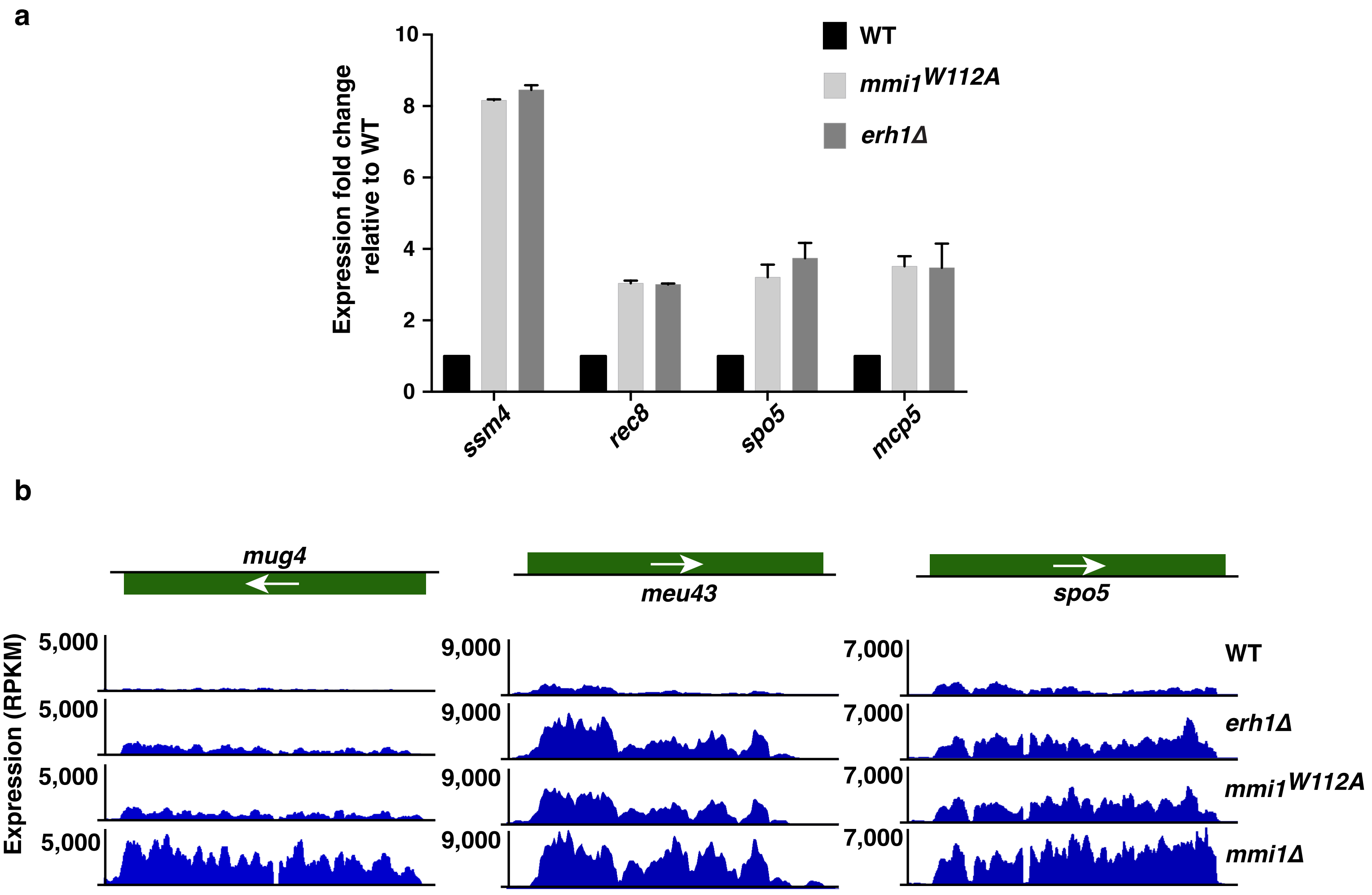

Supplementary Figure 6. Mmi1^{W112A} causes derepression of Mmi1-regulated loci. (a) RT-qPCR to measure RNA expression fold-change levels of *ssm4, rec8*, *spo5* and *mcp5* relative to control *leu1*. Shown are mean ± SEM for two independent biological experiments. **(b)** RNA-seq expression profiles of *mug4*, *meu43*, and *spo5* in WT, *erh1*Δ, *mmi1*^{W112A} and *mmi1*Δ. Shown is normalized RPKM expression.

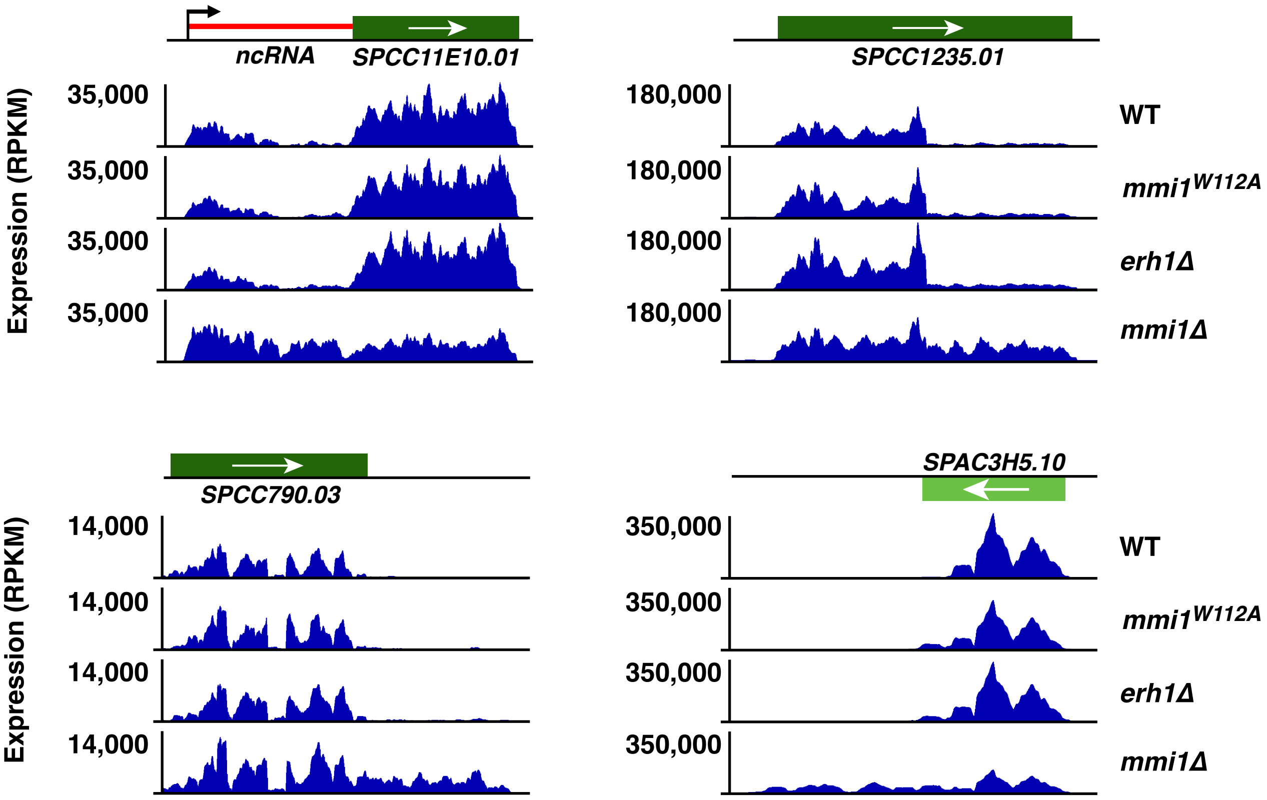

Supplementary Figure 7. Mmi1 controls transcript read-through independently from EMC. RNA-seq expression profiles of *SPCC11E10.01*, *SPCC1235.01*, *SPCC790.03*, and *SPAC3H5.10* loci in WT, *erh1*Δ, *mmi1*^{W112A} and *mmi1*Δ showing evidence of read-through predominantly in *mmi1*Δ. Shown is normalized RPKM expression.

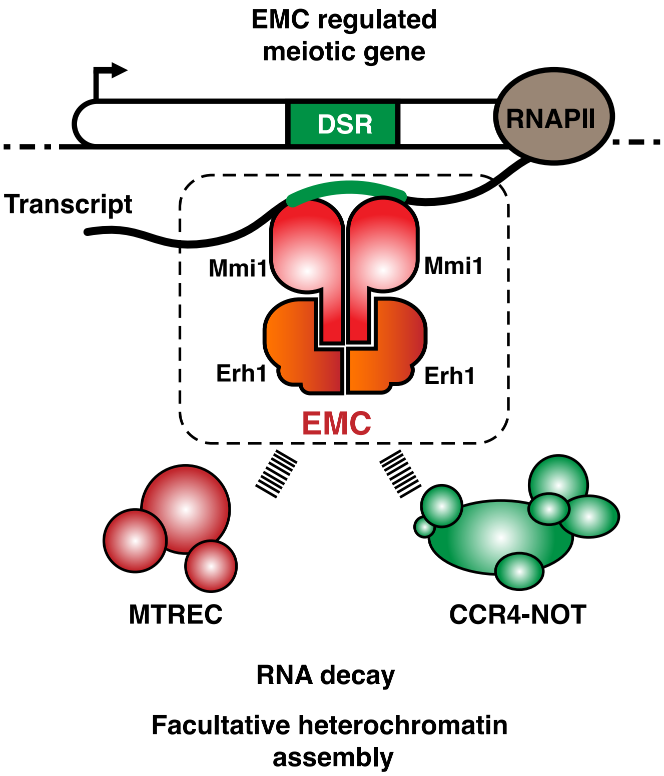

Supplementary Figure 8. The assembly of heterotetrameric complex EMC is critical for silencing meiotic genes. Mmi1 (red) binds to DSR-containing meiotic gene transcripts via its carboxy-terminal YTH domain but it engages Erh1 (orange) through its amino-terminal unstructured domain. Mmi1-Erh1 interaction involves the Erh1 dimer interface that is conserved in human ERH. EMC in turn associates with MTREC (dark red) and CCR4-NOT (green) protein complexes to trigger RNA degradation and facultative heterochromatin assembly, leading to silencing of target meiotic genes. Independently of EMC, Mmi1 also mediates transcription termination at meiotic genes and lncRNAs (not shown).

Supplementary Table 1 ITC results

Protein	Peptide	$\triangle H$ (kJ per mol)	$-T\triangle S$ (kJ per mol)	N	K_D uM
Erh1^{wt}	Mmi1$^{95\text{-}112}$	-106±3.02	72.3	0.90	0.833±0.194
Erh1^{wt}	Mmi1$^{95\text{-}111}$				N.D.
Erh1^{wt}	Mmi1$^{106\text{-}122}$	-73.6±2.69	45.5	0.89	9.94±1.19
Erh1^{wt}	Mmi1^{Y97A}	-46.1±1.20	13.8	1.27	1.75±0.317
Erh1^{wt}	Mmi1^{F99A}	-42.6±1.83	8.86	1.38	0.996±0.355
Erh1^{wt}	Mmi1^{S100A}	-36.0±3.16	6.04	1.36	4.73±1.62
Erh1^{wt}	Mmi1^{T104A}	-53.7±1.31	22.8	1.45	3.27±0.425
Erh1^{wt}	Mmi1^{Y106A}	-43.8±2.35	11.3	1.36	1.63±0.473
Erh1^{wt}	Mmi1^{H108A}	-9.97±2.43	-21.2	1.36	2.78±2.15
Erh1^{wt}	Mmi1^{Y110A}	-273±20.3	246	1	15.00±2.71
Erh1^{wt}	Mmi1^{W112A}				N.D.
Erh1^{wt}	Mmi1^{Y114A}	-61.8±1.07	31.1	0.97	3.52±0.323
Erh1^{wt}	Mmi1^{F115A}	-68.0±0.673	35.7	0.90	1.80±0.121
Erh1^{wt}	Mmi1^{R119A}	-46.3±1.28	13.1	1.12	1.22±0.27
Erh1 H9A	Mmi1^{wt}	41.8±0.974	9.13	1.32	1.53±0.205
Erh1 R23A	Mmi1^{wt}	-24.2±4.87	-8.55	1.28	1.48±1.07
Erh1 D27A	Mmi1^{wt}	-58.8±1.58	26.8	1.14	2.04±0.366
Erh1 D68A	Mmi1^{wt}	-47.6±1.30	17.2	1.34	3.96±0.551
Erh1 E85A	Mmi1^{wt}	-81.9±1.70	50.0	1.08	2.15±0.291

N.D. not detectable binding

Supplementary Table 2 Data collection and refinement statistics

	Erh1-Mmi1$^{95-122}$
Wavelength(Å)	0.978
Space group	$P3_221$
Cell parameters	
a, b, c (Å)	86.69, 86.69, 105.39
α, β, γ (°)	90, 90, 120
Resolution(Å)	40.00-2.70(2.85-2.70)[a]
R_{merge} (%)	10.5(133.6)
$I/\sigma I$	18.6(2.4)
Completeness (%)	100.0(100.0)
Average redundancy	15.8(14.5)
**Refinement**	
No. reflections (overall)	12998
No. reflections (test set)	708
R_{work}/R_{free}(%)	20.67/24.09
Number of atoms	
Erh1	1418
Mmi1	434
H_2O/SO_4^{2-}	12/25
B factors (Å^2)	
Erh1	74.16
Mmi1	78.77
H_2O/SO_4^{2-}	60.99/137.98
r.m.s. deviations	
Bond lengths (Å)	0.001
Bond angles (°)	0.384
Rampage plot % residues	
Favored	96.08
Allowed	3.92
Outliers	0

[a] Values in parentheses are for highest-resolution shell.

Supplementary Table 3. EMC-dependent and -independent heterochromatin islands

EMC-dependent heterochromatin islands

Island number*	Genomic coordinates	Nearby gene	H3K9me2 WT	H3K9me2 *erh1*Δ	H3K9me2 *mmi1*^{W112A}
1	chr1:578,438-581,236	*mcp7*	++++	-	-
2	chr1:2,445,561-2,447,627	*mug8*	+++	-	-
4	chr1:3,646,940-3,649,088	*spac8c9.04*	++++	-	-
5	chr1:3,727,304-3,729,052	*vps29*	++++	-	-
6	chr1:4,533,508-4,536,707	*ssm4*	++++	-	-
8	chr2:898,362-901,026	*mcp5*	+++	-	-
9	chr2:1,473,033-1,474,576	*mei4*	++++	-	-
16	chr2:3,626,236-3,628,856	*mbx2*	+++	-	-
20	chr3:2,367,827-2,369,841	*mug9*	++++	-	-

EMC-independent heterochromatin islands

3	chr1:2,520,728-2,523,306	*spac23h3.14*	++	++	++
7	chr1:4,653,094-4,654,219	*spac144.02*	++	++	++
13	chr2:1,868,149-1,868,769	*mug142*	++	+++	+++
14	chr2:2,198,013-2,200,240	*spncrna.394*	+++	+++	+++
15	chr2:2,338,199-2,341,250	*spbc24c6.09*	+++	+++	+++
19	chr3:1,037,820-1,039,129	*spcc1259.02c*	+++	+++	+++

*Heterochromatin island numbers correspond to those described previously[28]. For defining EMC-dependent and -independent heterochromatin islands, H3K9-dimethyl (H3K9me2) levels in WT, *erh1*Δ and *mmi1*^{W112A} cells were assessed by ChIP-Seq. Heterochromatin islands that showed a decrease in H3K9me2 levels in *mmi1*^{W112A} and *erh1*Δ, as compared to WT, are categorized as EMC-dependent, while others that showed no major changes in H3K9me levels are referred to as EMC-independent heterochromatin islands. Genomic coordinates correspond to *S. pombe* genome assembly v2.29.

Supplementary Table 4. List of strains and oligos used in this study

List of strains

**Name**	**Genotype**	**Source**
SPR854	*mat1Msmt0 leu1-32 ade-210 his2 ura4D18 mmi1::ura4+ mei4ΔC-myc<<natNT2*	lab stock
SPDF842	*matMstm0 leu1-32 ade6-210 his2 ura4D18 mmi1Δ::hphMX mei4ΔC-myc<<natNT2*	lab stock
SPT1414	*h90 leu1-32 ade6-216 mmi1-ts6::kanMX*	lab stock
SPT1369	*mat1Msmt0 leu1-32 ade6-210 his2 ura4D18 erh1Δ::kanMX mei4ΔC-myc<<natNT2*	lab stock
SPTV422	*mat1Msmt0 leu1-32 ade6-210 his2 ura4D18 mei4ΔC-myc<<natNT2*	this study
SPTV428	*mat1Msmt0 leu1-32 ade6-210 his2 ura4D18 mmi1(W112A) mei4ΔC-myc<<natNT2*	this study
SPTV482	*h90 leu1-32 ade6-216 erh1-GFP::kanMX mmi1(W112A) mei4ΔC-myc<<natNT2*	this study
SPTV485	*h90 leu1-32 ade6-216 erh1-GFP::kanMX mei4ΔC-myc<<natNT2*	this study
SPTV491	*mat1Msmt0 leu1-32 ade6-210 his2 ura4D18 FLAG-mmi1*	this study
SPTV492	*mat1Msmt0 leu1-32 ade6-210 his2 ura4D18 FLAG-mmi1(W112A)*	this study
SPTV506	*mat1Msmt0 leu1-32 ade6-210? his2 ura4D18 erh1-GFP::kanMX FLAG-mmi1*	this study
SPTV507	*mat1Msmt0 leu1-32 ade6-210? his2 ura4D18 erh1-GFP::kanMX FLAG-mmi1(W112A)*	this study
SPTV54	*mat1Msmt0 leu1-32 ade6-210 his2 ura4DS/E otr1R(Sph1)::ura4+*	this study
SPT1375	*h90 leu1-32 ade6-210 ura4D18 FLAG-mmi1*	lab stock
SPTV508	*h90 leu1-32 ade6-210? ura4D18 FLAG-mmi1(W112A)*	this study
SPT1194	*h90 leu1-32 ade6-210 ura4D18 erh1Δ::kanMX*	lab stock
SPNL202	*mat1Msmt0 leu1-32 ade6-210 his2 ura4DS/E or ura4-D18 pho1_ncRNA(-400/-1,200Δ::ura4)*	lab stock
SPTV536	*mat1Msmt0 leu1-32 ade6-216 his2 ura4DS/E or ura4-D18 pho1_ncRNA(-400/-1,200Δ::ura4) mmi1Δ::hphMX mei4ΔC-myc<<natNT2*	

List of oligos

**Name**	**Sequence**	**Purpose**
qPCR-leu1-fwd	CCTAAGGAGGCTGAAGCTATCG	qPCR
qPCR-leu1-rev	TCGCGAGTATAAAGACCACGTC	qPCR
mei4-qPCR-fwd	CATCGTTCGCACTCAACTGAC	qPCR
mei4-RTPCR-rev	GGTATCTCTCGCGTCGTCAAC	qPCR
ssm4-qPCR-fwd	CAGTTACTAATATCTTCTCAACCTG	qPCR
ssm4-qPCR-rev	GCACTGTTTAACTCGTCTATTAC	qPCR
ssm4-3end-qPCR-fwd	GGCTCAAACACAGTTTACGGG	qPCR
ssm4-3end-qPCR-rev	TCCTTGCAGGCAAAGGTCAA	qPCR
spo5-qPCR-fwd	GCCATTGGAGTCGAACACAG	qPCR
spo5-qPCR-rev	TGGCGTAGTACTTCTGCTGT	qPCR
rec8-2-qPCR-fwd	TACCGTTACCCGTTCCATTG	qPCR
rec8-2-qPCR-rev	CCATTGGGACAAAGTTCGAG	qPCR
mcp5-qPCR-fwd	TTGGGAGGCGTCAACTTATC	qPCR
mcp5-qPCR-rev	GAAATCGTCGTAGCCGTACTT	qPCR
AdapterPrimer-3RACE	GGCCACGCGTCGACTAGTACTTTTTTTTTTTTTTTTT	3' RACE
AUAP-3RACE	GGCCACGCGTCGACTAGTAC	3' RACE
ssm4-3R-AUAP-fwd1	CGATTCGTCTACTCCCACAAAGACAGCTAC	*ssm4* 3' RACE
ssm4-3R-AUAP-fwd2	AGCCAGGACAACTGGACTACACAAG	*ssm4* 3' RACE
VC512	GAGAAGAAGCGACCTGAGTTAAA	*leu1* 3' RACE
VC513	GTCGTCCTGAGCAAGGTTTAT	*leu1* 3' RACE
nam1F	GCTTCCATACCCCAACTTGA	Northern blot
nam1R	GATACGGTCGGCAATTCAAC	Northern blot
pho1Fwd1	ATTCCTTGGCTTTTTGGCCG	Northern blot
pho1T7Rev2	TAATACGACTCACTATAGGGCTGGGCGGCAGTGTAAATGT	Northern blot
ssm4_1	CCCGACGGACAAATAACTCA	smFISH
ssm4_2	CTAATTCTCCACGGATGAGG	smFISH
ssm4_3	TCAGTACTACCGGCAAATCG	smFISH
ssm4_4	GCCTAACCAAATACCACTTT	smFISH
ssm4_5	CATCATTTTTTCCTTTTCCA	smFISH
ssm4_6	AAATATCCCTTTTCCTTTCT	smFISH
ssm4_7	TCACATTCGAAGAGCATGCG	smFISH
ssm4_8	CTTGACGACACTTGGTCTTT	smFISH
ssm4_9	TTTCTGATCCTTTTTTCCTT	smFISH
ssm4_10	CCCTACGTGATTTTTGTTTA	smFISH
ssm4_11	TGTAATGCACAATGGGCGTG	smFISH
ssm4_12	TGTCTTTGTGGGAGTAGACG	smFISH
ssm4_13	ATTTGCTCGTATCTGAGTTT	smFISH
ssm4_14	AAACTACTATCGTCAAGCCC	smFISH
ssm4_15	TGCCATACAAATTCCTCTTC	smFISH
ssm4_16	TTTTTTTTCACATTCCTGCA	smFISH
ssm4_17	ACTTCCCTTTGAATGAGGAG	smFISH
ssm4_18	CCTTCCTTAATTCTGACTTT	smFISH
ssm4_19	AGGCGGTATTTTCACACATC	smFISH
ssm4_20	ACTGAGGTTCAACTTTCTCT	smFISH
ssm4_21	GCCTAAAATTCCGAGGTTTT	smFISH
ssm4_22	ATTTCTTTCTGCATAGCCAA	smFISH
ssm4_23	GCAACTTTCGCTTTCTTAGA	smFISH
ssm4_24	TGGCTTTTAGGAGTTTTGGA	smFISH
ssm4_25	TGTAACTTGTGTAGTCCAGT	smFISH
ssm4_26	CTTAGAAACCTCTGAGACCC	smFISH
ssm4_27	CTCAGTTATGTCGACCTGAA	smFISH
ssm4_28	AAATCCAAACTTCCCGGTAC	smFISH
ssm4_29	CTGTGCTATTCCAATGTACA	smFISH
ssm4_30	TCCACTGAAGAGTTGTTGGA	smFISH
ssm4_31	CAACAAAGCATTTTCCTCCA	smFISH
ssm4_32	TCTTGCTTCTATTTCAGTGT	smFISH
ssm4_33	CACCTCATTTAACTTTTCCG	smFISH
ssm4_34	ACTGAGATAGCCTTTGAGTT	smFISH
ssm4_35	CTTCTGTCAAAGCAGGTTGA	smFISH
ssm4_36	ATTCCTCTTGAGAGTCTGAT	smFISH
ssm4_37	TTGCTTTCTCAAGTTTTGCA	smFISH
ssm4_38	TTGCACTGTTTAACTCGTCT	smFISH
ssm4_39	TCCTCCACTTGTTTTGATAG	smFISH
ssm4_40	AGGTCGTCTTTCATACTACT	smFISH
ssm4_41	ATATTTATCGGCCAGTTTCA	smFISH
ssm4_42	CACTAGTATTGTCACCTTGT	smFISH
ssm4_43	AAACTGTGTTTGAGCCAGTC	smFISH
ssm4_44	GGGCTTGGAATTGAGTTTTC	smFISH
ssm4_45	ATTTTCTTATCTCCTTGCAG	smFISH
ssm4_46	GCTTTTCTACTGAAACGGGT	smFISH
ssm4_47	TTTTTCCAATTCAAGGCCAG	smFISH
ssm4_48	GATGAAAACTGGGTCGCTCG	smFISH
