# Supplementary material for: A conserved dimer interface connects ERH and YTH family proteins to promote gene silencing
Source: Nat Commun. 2019 Jan 16;10:251. doi: 10.1038/s41467-018-08273-9 (PMC6335422; doi:10.1038/s41467-018-08273-9)
Supplement: Supplementary file 3 — Reporting Summary [file 41467_2018_8273_MOESM3_ESM.pdf]

nature research

Corresponding author(s): Fudong Li and Shiv Grewal

Last updated by author(s): Dec 15, 2018

Reporting Summary

Nature Research wishes to improve the reproducibility of the work that we publish. This form provides structure for consistency and transparency in reporting. For further information on Nature Research policies, see Authors & Referees and the Editorial Policy Checklist.

Statistics

For all statistical analyses, confirm that the following items are present in the figure legend, table legend, main text, or Methods section.

n/a	Confirmed	
☐	☒	The exact sample size (*n*) for each experimental group/condition, given as a discrete number and unit of measurement
☒	☐	A statement on whether measurements were taken from distinct samples or whether the same sample was measured repeatedly
☒	☐	The statistical test(s) used AND whether they are one- or two-sided<br>*Only common tests should be described solely by name; describe more complex techniques in the Methods section.*
☒	☐	A description of all covariates tested
☒	☐	A description of any assumptions or corrections, such as tests of normality and adjustment for multiple comparisons
☐	☒	A full description of the statistical parameters including central tendency (e.g. means) or other basic estimates (e.g. regression coefficient) AND variation (e.g. standard deviation) or associated estimates of uncertainty (e.g. confidence intervals)
☒	☐	For null hypothesis testing, the test statistic (e.g. *F*, *t*, *r*) with confidence intervals, effect sizes, degrees of freedom and *P* value noted<br>*Give P values as exact values whenever suitable.*
☒	☐	For Bayesian analysis, information on the choice of priors and Markov chain Monte Carlo settings
☒	☐	For hierarchical and complex designs, identification of the appropriate level for tests and full reporting of outcomes
☒	☐	Estimates of effect sizes (e.g. Cohen's *d*, Pearson's *r*), indicating how they were calculated

Our web collection on statistics for biologists contains articles on many of the points above.

Software and code

Policy information about availability of computer code

Data collection	Not applicable
Data analysis	ChIP-seq alignment was performed using BWA-MEM and downstream processing using modules correctGCBias and BamCompare from the Deeptools tool-suite. RNA-seq alignment was performed using Tophat2; differential-expression analyses were performed using Cufflinks. No custom algorithms or software were used in this study.

For manuscripts utilizing custom algorithms or software that are central to the research but not yet described in published literature, software must be made available to editors/reviewers. We strongly encourage code deposition in a community repository (e.g. GitHub). See the Nature Research guidelines for submitting code & software for further information.

Data

Policy information about availability of data

All manuscripts must include a data availability statement. This statement should provide the following information, where applicable:

- Accession codes, unique identifiers, or web links for publicly available datasets
- A list of figures that have associated raw data
- A description of any restrictions on data availability

The atomic coordinates and structure factors for the EMC complex have been deposited to the Protein Data Bank (PDB) under the accession code PDB 6AKJ. Genomic datasets are deposited in the Gene Expression Omnibus with accession numbers GSE119604 and GSE119605. All other materials are available from the corresponding authors upon reasonable request. Uncropped gel images and other source data underlying Figs 1b-d, 3d, 4a, b and e, 6a, 8a, c and d are provided as a Source Data file. A reporting summary for this article is available as a Supplementary Information file.

Field-specific reporting

Please select the one below that is the best fit for your research. If you are not sure, read the appropriate sections before making your selection.

☒ Life sciences ☐ Behavioural & social sciences ☐ Ecological, evolutionary & environmental sciences

For a reference copy of the document with all sections, see nature.com/documents/nr-reporting-summary-flat.pdf

Life sciences study design

All studies must disclose on these points even when the disclosure is negative.

Sample size	Sample sizes were chosen as 2 or greater except to reproduce results which have already been published, in which case, 1 replicate was performed to maintain experimental consistency with the rest of this study.
Data exclusions	No data was excluded.
Replication	All attempts at replication of the results in this study were successful.
Randomization	Samples were allocated into experimental groups based on sample genotypes.
Blinding	Blinding is not relevant to this study because this is a prospective study where samples are allocated based on known genotypes.

Reporting for specific materials, systems and methods

We require information from authors about some types of materials, experimental systems and methods used in many studies. Here, indicate whether each material, system or method listed is relevant to your study. If you are not sure if a list item applies to your research, read the appropriate section before selecting a response.

Materials & experimental systems

n/a	Involved in the study	
☐	☒	Antibodies
☐	☒	Eukaryotic cell lines
☒	☐	Palaeontology
☒	☐	Animals and other organisms
☒	☐	Human research participants
☒	☐	Clinical data

Methods

n/a	Involved in the study	
☐	☒	ChIP-seq
☒	☐	Flow cytometry
☒	☐	MRI-based neuroimaging

Antibodies

Antibodies used	ChIP experiments were performed using anti-H3K9me2 (ab115159, Abcam) and anti-GFP (ab290, Abcam); immunoprecipitation and western blots were performed using anti-GFP (7.1 and 13.1, Roche and GTA20, Chromotek), anti-FLAG (F3165 , Sigma), and anti-Cdc2 (Y100.4, Santa Cruz).
Validation	Anti-GFP and anti-FLAG antibodies were tested for their affinity to known substrates by western blotting. Anti-H3K9me2 antibodies have been extensively used in our lab and enrichment at known loci were determined by ChIP-seq and ChIP-qPCR.

Eukaryotic cell lines

Policy information about cell lines

Cell line source(s)	Lab stocks
Authentication	Cells were authenticated by PCR analyses and/or Sanger sequencing.
Mycoplasma contamination	No testing for mycoplasma because cells are yeast.
Commonly misidentified lines (See ICLAC register)	Not applicable

ChIP-seq

Data deposition

☒ Confirm that both raw and final processed data have been deposited in a public database such as GEO.

☒ Confirm that you have deposited or provided access to graph files (e.g. BED files) for the called peaks.

Data access links<br>*May remain private before publication.*	Data related to GEO accession number GSE119604 can be found at https://www.ncbi.nlm.nih.gov/geo/query/acc.cgi?acc=GSE119604
Files in database submission	GSM3378732_wt-1-k9-ratio.bw<br>GSM3378733_wt-2-k9-ratio.bw<br>GSM3378734_w112a-1-k9-ratio.bw<br>GSM3378735_w112a-2-k9-ratio.bw<br>GSM3378736_erh1d-1-k9-ratio.bw
Genome browser session<br>(e.g. UCSC)	Not applicable

Methodology

Replicates	Two independent biological replicates for WT and mmi1(W112A) samples. One replicate for erh1 deletion sample because ChIP data for this sample has been published elsewhere (Sugiyama et al. 2016).
Sequencing depth	All sequencing were performed as multiplexed single-end 75-nt runs. For each sample, 31-35 million reads were obtained for analyses.
Antibodies	Anti-H3K9me2 antibodies (ab115159, Abcam) were used.
Peak calling parameters	ChIP aligned data were first normalized to an average 1X coverage, then ratios relative to input were computed per ChIP sample.
Data quality	FastQC was used to assess sequencing quality of data; positive and negative control regions were used to ensure high-quality of the processed data; quantitative PCR experiments were independently performed to validate several observed regions-of-interest.
Software	Alignments were performed using BWA-MEM aligner. Aligned data were processed using correctGCBias and BamCompare modules from the Deeptools tool-suite.
